# High Inflammatory Burden: A Potential Cause of Myocardial Injury in Critically Ill Patients With COVID-19

**DOI:** 10.3389/fcvm.2020.00128

**Published:** 2020-07-07

**Authors:** Yanjun Song, Peng Gao, Tian Ran, Hao Qian, Fan Guo, Long Chang, Wei Wu, Shuyang Zhang

**Affiliations:** ^1^Department of Cardiology, Chinese Academy of Medical Sciences and Peking Union Medical College, Beijing, China; ^2^Department of Internal Medicine, Chinese Academy of Medical Sciences and Peking Union Medical College, Beijing, China

**Keywords:** COVID-19, critical patients, myocardial injury, inflammation, In-ICU mortality

## Abstract

**Background:** Myocardial injury is a severe complication of novel coronavirus disease (COVID-19), and inflammation has been suggested as a potential cause of myocardial injury. However, the correlation of myocardial injury with inflammation in COVID-19 patients has not been revealed so far.

**Method:** This retrospective single-center cohort study enrolled 64 critically ill patients with COVID-19. Patients were categorized into two groups by the presence of myocardial injury on admission. Demographic data, clinical characteristics, laboratory tests, treatments, and outcomes were analyzed in this study.

**Result:** Of these patients, the mean age was 64.8 ± 12.2 years old, and 34 (53.1%) were diagnosed with myocardial injury. Compared with non-myocardial injury patients, myocardial injury patients were older (67.8 ± 10.3 vs. 61.3 ± 13.3 years; *P* = 0.033), had more cardiovascular (CV) risk factors such as smoking (16 [47.06%] vs. 7 [23.33%]; *P* = 0.048) and were more likely to develop CV comorbidities (13 [38.2%] vs. 2 [6.7%]; *P* = 0.003). Scores on the Acute Physiology and Chronic Health Evaluation II (median [interquartile range (IQR)] 19.0 [13.25–25.0] vs. 13.0 [9.25-18.75]; *P* = 0.005) and Sequential Organ Failure Assessment systems (7.0 [5.0–10.0] vs. 4.5 [3.0–6.0]; *P* < 0.001) were significantly higher in the myocardial injury group. In addition, patients with myocardial injury had higher mortality than those without myocardial injury (29 [85.29%] vs. 18 [60.00%]; *P* = 0.022). Cox regression suggested that myocardial injury was an independent risk factor for high mortality during the time from admission to death (hazard ratio [HR], 2.06 [95% confidence interval (CI), 1.10–3.83]; *P* = 0.023). Plasma levels of high-sensitivity C-reactive protein (hs-CRP), interleukin (IL)-1β, interleukin-2 receptor (IL-2R), IL-6, IL-8, IL-10, and tumor necrosis factor-α (TNF-α) exceeded the normal limits, and levels of hs-CRP, IL-2R, IL-6, IL-8, and TNF-α were statistically higher in the myocardial injury group than in the non-myocardial injury group. Multiple-variate logistic regression showed that plasma levels of hs-CRP (odds ratio [OR] 6.23, [95% CI, 1.93–20.12], *P* = 0.002), IL-6 (OR 13.63, [95% CI, 3.33–55.71]; *P* < 0.001) and TNF-α (OR 19.95, [95% CI, 4.93–80.78]; *P* < 0.001) were positively correlated with the incidence of myocardial injury.

**Conclusion:** Myocardial injury is a common complication that serves as an independent risk factor for a high mortality rate among in-ICU patients with COVID-19. A high inflammatory burden may play a potential role in the occurrence of myocardial injury.

## Introduction

Coronavirus disease 2019 (COVID-19), a novel coronavirus–infected pneumonia caused by severe acute respiratory syndrome coronavirus 2 (SARS-CoV-2), has currently become a severe global health problem (1, 2). Myocardial injury was suggested to be prevalent in COVID-19 patients, which has contributed to fatal complications and high mortality rates (3–5). However, the mechanism underlying myocardial injury has not yet been confirmed. Recently, several studies revealed that COVID-19 patients were mostly in a high systemic inflammatory status with severe cytokine storms (e.g., high levels of interleukin [IL]-6, IL-8, and tumor necrosis factor-α [TNF-α]), which contributed to fatal complications (6–8). Given that inflammation has been revealed as a great contributor to all forms of myocardial injury (9), COVID-19-induced systemic inflammation was suggested to potentially cause myocardial injury in COVID-19 patients. In this study, we aimed to investigate the association of inflammation with myocardial injury in critically ill patients with COVID-19.

## Materials and Methods

### Study Design and Participants

This single-center, retrospective, observational study was performed in a newly built intensive care unit (ICU) of Tongji Hospital (Sino-French New City Campus), Huazhong University of Science and Technology, Wuhan, China. This ICU was designated to treat critically ill patients with COVID-19. We retrospectively analyzed 64 COVID-19 patients admitted to the ICU in this study. The data cut-off for investigation of survival status was March 26, 2020. All the patients were confirmed as COVID-19 with a positive result on real-time reverse-transcriptase–polymerase-chain-reaction (RT-PCR) assay of throat-swab specimens. The study protocol conformed to the ethical guidelines of the 1975 Declaration of Helsinki and was approved by the Ethics Committee of PUMC Hospital. Written informed consent was waived due to the rapid emergence of this infectious disease. No potentially identifiable human images or data is presented in this study. Plasma levels of inflammatory cytokines test were finished within 24 h when patients were admitted. All the other laboratory tests were finished within 6 h after admission. All the data included in this study were part of routine patient care in ICU.

### Data Collection and Study Design

The data collected in this study were extracted from electronic medical records reviewed by the clinical team from Peking Union Medical College Hospital (PUMCH). Patient data included demographics, survival time from ICU admission to death, baseline characteristics (i.e., prior medical illness, cardiovascular risk factors), in-ICU clinical information (i.e., vital signs, complications, and therapeutic measures), laboratory results and outcomes. We also documented patients' Acute Physiology and Chronic Health Evaluation II (APACHE II) and Sequential Organ Failure Assessment (SOFA) scores on admission to the ICU.

### Outcome

Patients were categorized into two groups (myocardial injury vs. non-myocardial injury) based on their on-admission high-sensitivity cardiac troponin I (hs-cTnI) levels. The primary outcome was 28-day mortality after ICU admission. myocardial injury was defined as an elevated cardiac troponin value above the 99th percentile of the upper reference limit (34.2 ng/ml) according to the fourth Universal Definition of Myocardial Infarction (10). Prior cardiovascular (CV) disease was defined as coronary artery disease (CAD), myocardial infarction, heart failure or stroke, and in-ICU CV complications were defined as arrythmias (atrial tachycardia, atrial fibrillation, ventricular tachycardia, and/or ventricular fibrillation), cardiac arrest, cardiac shock or myocardial infarction.

Acute respiratory distress syndrome (ARDS) and acute kidney injury (AKI) were diagnosed according to the Berlin Definition and KDIGO clinical practice guidelines, respectively (11, 12).

### Statistical Analysis

Categorical variables were presented as counts and percentages. Continuous variables were described as means ± standard deviations (SDs) for normally distributed data and medians (interquartile ranges [IQRs]) for non-normally distributed data. A two-sample *T*-test was used to assess whether there were significant differences in continuous variables when they were normally distributed; otherwise, the Mann-Whitney U-test was used. The χ^2^ test was applied to test the differences in categorical variables, although Fisher's exact test was used for comparisons with small sample sizes. Kaplan-Meier (K-M) plots and Cox proportional hazards regression models were used for survival analysis, which was based on the time from ICU admission to death. The log-rank test was used to confirm the differences between K-M plots. Logistics regression was applied to test the contribution of inflammation to the incidence of myocardial injury. Concretely, we firstly involve single variate into logistics regression, and then put the variates with *P* < 0.05 into regression equation thereby giving the final result. Statistical significance was determined when two-sided α was <0.05. All statistical analyses were performed using SPSS 21.0 software (IBM, Armonk, NY).

## Results

### General Characteristics of Critically Ill Patients With COVID-19

**Figure 1:**
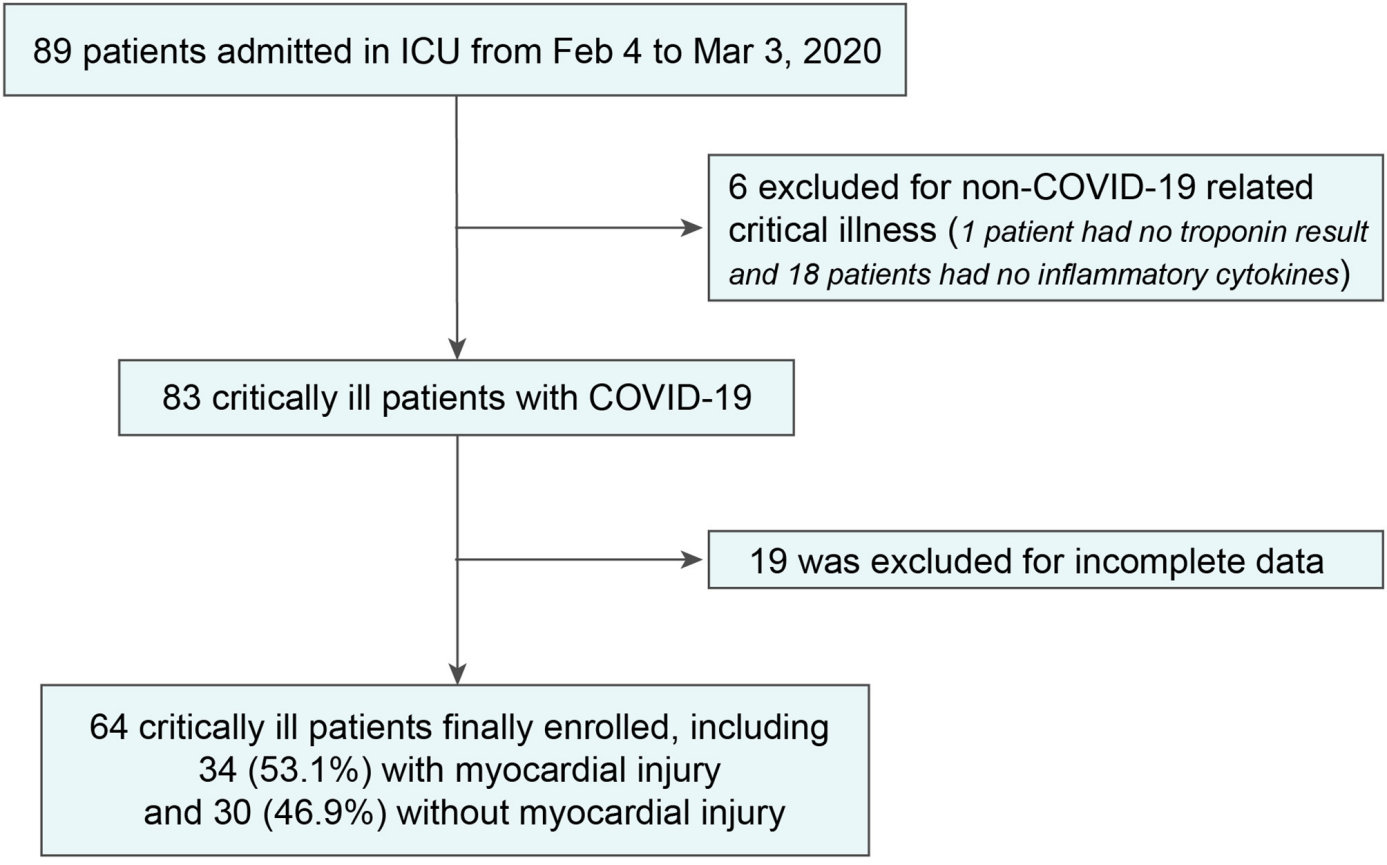
Study flow diagram. ICU, intensive care unit; COVID-19, novel coronavirus disease.

Night-nine adults admitted to the ICU from February 4 to March 3, 2020, were studied. After excluding 6 patients who were not admitted for COVID-19-related critical illness and 19 patients with incomplete data (1 patient had no troponin result and 18 patients had no inflammatory cytokines), we included 64 in-ICU patients in the final analysis (Figure 1).

Of these patients, 42 (65.6%) were men, the mean age was 64.8 ± 12.2 years (range, 26–92 years), and 47 patients reached the primary endpoint during the follow-up time. Prior CV diseases and CV risk factors were common in critical patients, as there were 13 patients (20.3%) with pre-existing CV diseases (CAD: 7 [10.9%]; heart failure: 2 [3.1%]; stroke: 8 [12.5%]) and 43 (67.2%) patients with 1 or more coexisting CV risk factors (hypertension: 35 [54.7%]; diabetes: 15 [23.4%]; smoking: 23 [35.9%]). ARDS was the most common in-ICU complication (62 [96.88%]), followed by AKI (21 [32.8%]) and CV complications (15 [23.4%]). Laboratory results showed that coagulation dysfunction and high inflammatory burden were common in these critical patients, as most coagulation indicators and inflammatory indicators were higher than the normal limits. In addition, plasma levels of N-terminal pro-B-type natriuretic peptide (NT-proBNP) and hs-cTnI were also significantly increased (Table 2). Fifty-two (81.3%) patients received invasive mechanical ventilation, and 19 (29.7%) received non-invasive mechanical ventilation. Immune therapies were commonly used in critical patients (glucocorticoids: 54 [84.4%]; tocilizumab: 7 [10.94%]). More detailed information is presented in Tables 1, 2.

**Table 1:** Demographics and clinical characteristics of critically ill patients with COVID-19.

	**Myocardial injury (*n =* 34)**	**Non-myocardial injury (*n =* 30)**	**Total (*n =* 64)**	***P*-value**
**Age (yrs)**	67.8 ± 10.3	61.3 ± 13.3	64.8 ± 12.2	0.033
**Male**	24 (70.6%)	18 (60.0%)	42 (65.6%)	0.37
**Prior CV diseases**				
CAD	6 (17.7%)	1 (3.3%)	7 (10.9%)	0.11
Heart failure	2 (5.9%)	0 (0.00%)	2 (3.1%)	0.49
Stroke	6 (17.7%)	2 (6.7%)	8 (12.5%)	0.27
**CV risk factors**				
Hypertension	22 (64.7%)	13 (43.3%)	35 (54.7%)	0.087
Diabetes	10 (29.4%)	5 (16.7%)	15 (23.4%)	0.23
Smoking	16 (47.1%)	7 (23.3%)	23 (35.9%)	0.048
**Vital signs**				
Fever	12 (35.3%)	14 (46.7%)	26 (40.6%)	0.36
HR (bpm)	112.9 ± 20.4	106.7 ± 18.5	110.0 ± 19.6	0.21
SBP (mmHg)	124.6 ± 26.3	127.8 ± 20.5	126.1 ± 23.6	0.60
DBP (mmHg)	74.8 ± 14.5	77.7 ± 14.2	76.2 ± 14.4	0.42
RR (times/min)	29.3 ± 8.8	27.5 ± 7.1	28.4 ± 8.0	0.38
**Critical score**				
APACHE II score^*^	19.0 (13.3–25.0)	13.0 (9.3–18.8)	15.0 (12.0–22.0)	0.005
SOFA score^*^	7.0 (5.0–10.0)	4.5 (3.0–6.0)	6.0 (4.0–8.0)	<0.001
**Complications**				
CV complications	13 (38.2%)	2 (6.7%)	15 (23.4%)	0.003
ARDS	34 (100.0%)	28 (93.3%)	62 (96.9%)	0.22
AKI	13 (38.2%)	8 (26.7%)	21 (32.8%)	0.33
Live dysfunction	6 (17.7%)	10 (33.3%)	16 (25.0%)	0.15
**Symptom onset to ICU admission (days)**	15.0 (11.0–23.0)	16.5 (9.3–23.5)	15.5 (10.0–23.3)	0.91
**In-ICU therapy**				
Non-invasive mechanical ventilation	12 (35.3%)	7 (23.3%)	19 (29.7%)	0.30
Invasive mechanical ventilation	26 (76.5%)	26 (86.7%)	52 (81.5%)	0.30
Immunoglobulin	26 (76.5%)	25 (83.3%)	51 (79.7%)	0.50
Glucocorticoids	26 (76.5%)	28 (93.3%)	54 (84.4%)	0.064
Vasoconstrictive agents	24 (70.6%)	18 (60.0%)	42 (65.6%)	0.37
Tocilizumab	2 (5.9%)	5 (16.7%)	7 (10.9%)	0.17
**Death**				
All-cause death	29 (85.3%)	18 (60.0%)	47 (73.4%)	0.022
Survival time^*^	7.0 (3.0–13.75)	19.0 (10.0–38.75)	11.5 (5.0-35.0)	0.002

* *Continuous variables with non-normal distribution presented as “median (IQR).” CV, cardiovascular; CAD, coronary artery disease; ICU, intensive care unit. HR, heart rate; RR, respiratory rate; SBP, systolic blood pressure; DBP, diastolic blood pressure; APACHE II, Acute Physiology and Chronic Health Evaluation II; SOFA, Sequential Organ Failure Assessment; AKI, acute kidney injury; ARDS, acute respiratory distress syndrome. P-values present the differences between myocardial injury and non-myocardial injury patients*.

**Table 2:** Laboratory tests between COVID-19 patients with and without myocardial injury.

	**Normal range**	**Myocardial injury (*n =* 34)**	**Non-myocardial injury (*n =* 30)**	**Total (*n =* 64)**	***P*-value**
White blood count, ×10^9^/L	3.5–9.5	12.5 ± 5.1	11.1 ± 5.7	11.9 ± 5.4	0.30
Neutrophils^*^ (%)	40.0–75.0	91.7 (88.7–95.1)	90.7 (83.2–93.4)	91.1 (85.9–94.0)	0.05
Lymphocytes, ×10^9^/L	1.1–3.2	0.5 ± 0.4	0.7 ± 0.4	0.6 ± 0.4	0.25
Hemoglobin, g/L	130.0–175.0	121.8 ± 21.7	123.0 ± 19.8	122.4 ± 20.7	0.82
Platelets, ×10^9^/L	125.0–350.0	155.0 ± 89.4	197.0 ± 105.6	174.7 ± 98.8	0.09
ALT^*^, U/L	≤41.0	26.0 (14.0–41.0)	27.5 (22.0–36.8)	29.0 (19.8–42.0)	0.89
Total bilirubin^*^, μmol/L	≤26.0	13.2 (9.6–21.2)	14.5 (8.0–18.7)	13.7 (8.7–19.0)	0.40
Albumin g/L	35.0–52.0	28.0 ± 4.3	30.5 ± 6.1	29.2 ± 5.3	0.065
Creatinine^*^, μmol/L	59.0–104.0	88.5 (71.5–124.0)	67.0 (48.5–86.0)	81.0 (58.0–107.8)	0.005
BUN^*^, mmol/L	3.6–9.5	10.2 (7.1–20.7)	7.1 (5.4–10.3)	7.8 (6.3–14.4)	0.013
Serum potassium, mmol/L	3.5–5.1	4.5 ± 0.8	4.5 ± 1.0	4.5 ± 0.9	0.84
PT^*^, s	11.5–14.5	17.3 (15.7–18.2)	15.4 (14.7–16.3)	16.15 (15.0–17.6)	0.005
APTT^*^, s	29.0–42.0	41.8 (38.4–45.3)	41.5 (37.4–45.1)	41.6 (37.5–45.2)	0.68
INR^*^	0.8–1.2	1.4 (1.2–1.5)	1.2 (1.1–1.3)	1.3 (1.2–1.4)	0.002
Fbg, g/L	2.0–4.0	4.5 ± 3.9	4.6 ± 2.1	4.5 ± 3.2	0.29
D-dimer^*^, mg/L	<0.5	21.0 (7.5–21.0)	3.7 (1.9–21.0)	14.7 (2.8–21.0)	0.005
hsCRP^*^, mg/L	<1.0	155.0 (78.3–210.9)	45.0 (16.0–96.0)	86.5 (34.7–194.3)	<0.001
IL1 β, pg/ml	<5.0	6.5 ± 4.4	5.2 ± 0.7	5.9 ± 3.3	0.53
IL2 R^*^, pg/ml	223.0–710.0	1152.0 (741.0–1679.0)	731.0 (302.0–1224.5)	1041.0 (554.3–1485.3)	0.02
IL-6, pg/ml	<7.0	982.2 ± 1517.9	204.4 ± 400.3	617.6 ± 1197.4	0.008
IL-8^*^, pg/ml	<62.0	48.5 (21.1–156.1)	22.7 (14.4–42.9)	29.4 (18.1–76.7)	0.015
IL-10^*^, pg/ml	<9.1	10.7 (6.3–24.0)	10.5 (5.1–15.5)	10.7 (5.5–19.6)	0.30
TNF-α^*^, pg/ml	<8. 1	19.8 (14.7–40.1)	9.0 (7.1–11.0)	13.8 (9.3–23.0)	<0.001
HscTnI^*^, ng/L	≤34.2	276.1 (139.1–909.7)	12.1 (4.7–18.9)	46.5 (12.1–374.1)	<0.001
NT-proBNP^*^, ng/L	<241.0	1947.5 (644.8–4393.5)	372.0 (73.8–836.5)	816.5 (254.5–2585.0)	<0.001

* *Continuous variables with non-normal distribution presented as “median (IQR).” ALT, alanine aminotransferase; BUN, blood urea nitrogen; hsCRP, high-sensitivity C-reative protein; IL, interleukin; IL-2R, interleukin-2 receptor; TNF-α, tumor necrosis factor α; PT, prothrombin time; APTT, activated partial thromboplastin time; INR, international normalized ratio; Fbg, fibrinogen; hs-cTnI, high-sensitive cardiac troponin I; NT-proBNP, N-terminal pro-B-type natriuretic peptide. P-values present the differences between MI and non-MI patients*.

### Differences Between Myocardial Injury Patients and Non-myocardial Injury Patients

In our study, 34 patients (53.1%) were diagnosed with myocardial injury. Compared with non-myocardial injury patients, the myocardial injury patients were significantly older (67.8 ± 10.3 vs. 61.3 ± 13.3 years; *P* = 0.033), more likely to have preexisting cardiovascular diseases (13 [38.2%] vs. 3 [10.0%]; *P* = 0.009), and had more CV risk factors (smoking: 16 [47.1%] vs. 7 [23.3%]; *P* = 0.048) and CV comorbidities (13 [38.2%] vs. 2 [6.7%]; *P* = 0.003) (Table 1). Concomitantly, patients with myocardial injury had higher APACHE II (19.0 [13.25–25.0] vs. 13.0 [9.25–18.75]; *P* = 0.005) and SOFA system scores than those of the non-myocardial injury group (7.0 [5.0–10.0] vs. 4.5 [3.0–6.0]; *P* < 0.001).

Regarding laboratory results, myocardial injury patients showed significant increases in the plasma levels of creatinine, blood urea nitrogen, D-dimer, high-sensitivity C-reactive protein (hs-CRP) (155.0 [78.3–210.9] vs. 45.0 [16.0–96.0] mg/L; *P* < 0.001), interleukin-2 receptor (IL-2R) (1152.0 [741.0–1679.0] vs. 731.0 [302.0–1224.5] pg/ml; *P* = 0.02), IL-6 (1144.7 ± 1466.7 vs. 204.4 ± 400.3 pg/ml; *P* < 0.001), IL-8 (48.5 [21.1–156.1] vs. 22.7 [14.4–42.9] pg/ml; *P* = 0.015) and TNF-α (19.8 [14.7–40.1] vs. 9.0 [7.1–11.0] pg/ml; *P* < 0.001) (Table 2). In addition, the levels of creatinine, blood urea nitrogen and D-dimer were also significantly increased in the myocardial injury group. However, no significant differences were found in the applications of in-ICU therapies between myocardial injury and non-myocardial injury patients (Table 1).

**Figure 2:**
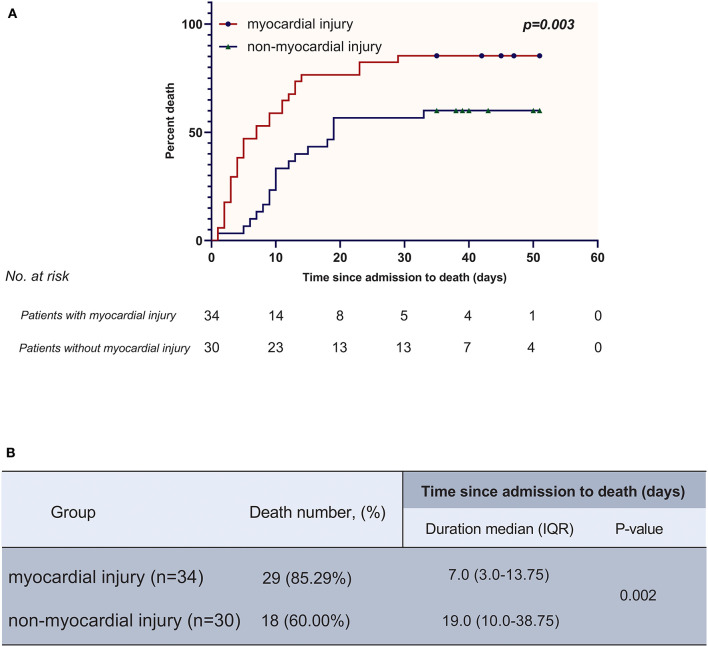
**(A)** K-M plot for patients with myocardial injury and without myocardial injury. **(B)** Comparisons of death number and time since admission to death between myocardial injury group and non-myocardial injury group.

In the survival analysis, the mortality rate was much higher in the myocardial injury group than in the non-myocardial injury group (29 [85.29%] vs. 18 [60.00%]; *P* = 0.022). Furthermore, myocardial injury was demonstrated as an independent risk factor for reduced survival time from admission to death (hazard ratio [HR], 2.06 [95% confidence interval (CI), 1.10–3.83]; *P* = 0.023) by a multivariable adjusted Cox proportional hazard regression model adjusting for age, smoking history and pre-existing with CVD. The high mortality in the myocardial injury group was also shown in the K-M survival curves (log-rank test, *P* = 0.003) (Figure 2).

### Association of High Inflammatory Burden With the Incidence of Myocardial Injury in Critically Ill Patients With COVID-19

**Figure 3:**
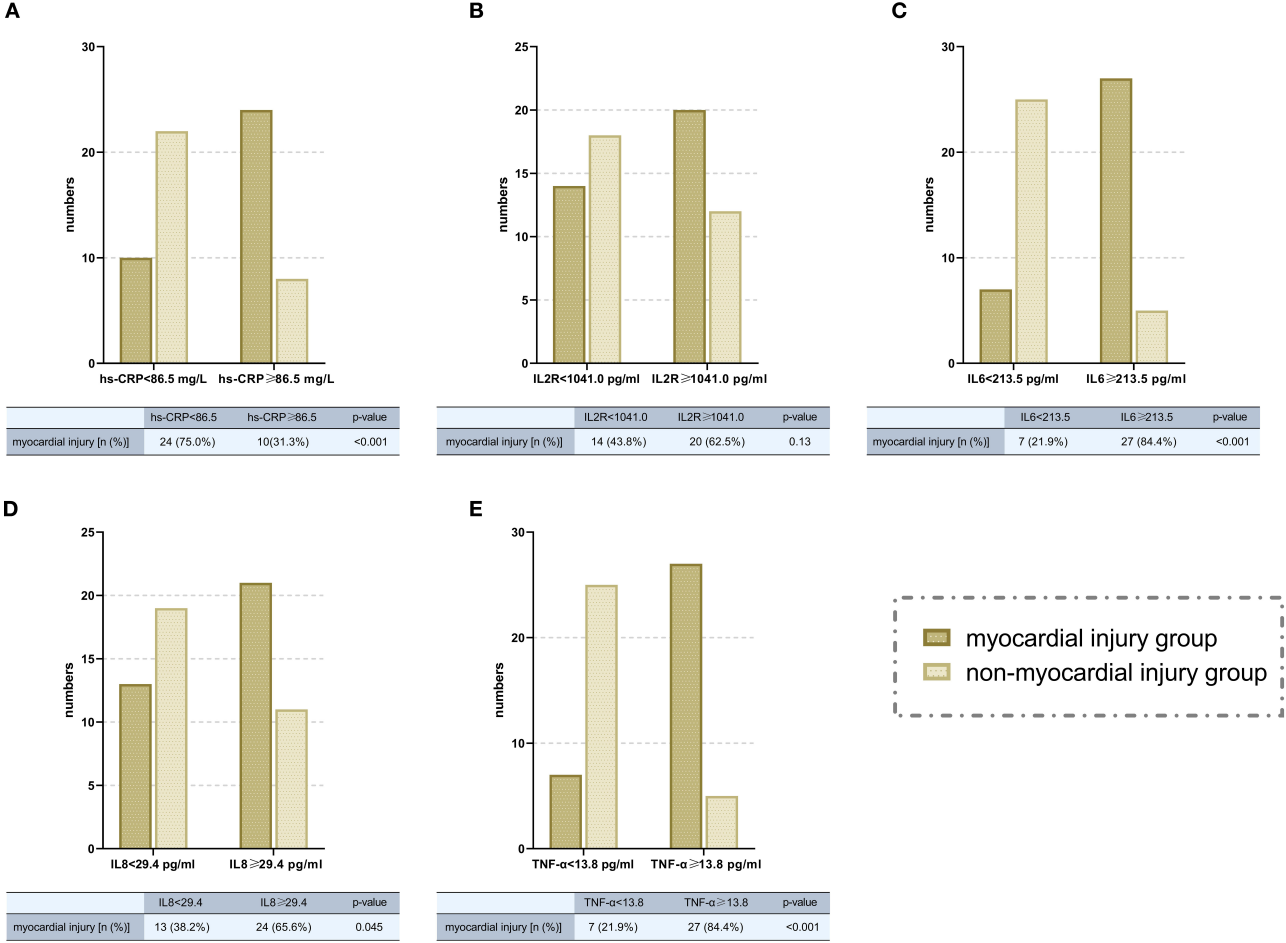
Comparisons of the numbers of patients with myocardial injury in high/low inflammatory burden groups (divided according to the cut-off of median levels of different inflammatory cytokines. **A**, hs-CRP; **B**, IL-2R; **C**, IL-6; **D**, IL-8; **E**, TNF-α). hsCRP, high-sensitivity C-reative protein; IL, interleukin; IL-2R, interleukin-2 receptor; TNF-α, tumor necrosis factor α; OR, odds ratio; CI, confidence interval.

Most inflammatory biomarkers were significantly higher in COVID-19 patients with myocardial injury than in those without myocardial injury (Table 2). Consistently patients with higher inflammatory burden (plasma levels of inflammatory cytokines higher than the median levels) were also more likely to develop myocardial injury (Figure 3). To investigate the relation between high inflammatory burden with myocardial injury, we set the dependent variable to “myocardial injury” and set independent variables to the high/low inflammatory burden which was divided according to the cut-off of the median levels of inflammatory cytokines. In the univariate logistic regression analysis, we found that high plasma levels (higher than the median levels) of high-sensitivity C-reactive protein (hs-CRP) (odds ratio [OR] 10.80, [95% CI, 1.97–59.15]; *P* = 0.006), IL-6 (OR 9.13, [95% CI, 2.92–28.50]; *P* < 0.001), IL-8 (OR 7.27, [95% CI, 1.35–39.05]; *P* = 0.021) and TNF-α (OR 17.36, [95% CI, 3.04–99.20]; *P* = 0.001) were positively associated with the incidence of myocardial injury. We further entered these biomarkers into the multivariate logistic regression with adjusting variates of age, smoking history and pre-existing with CVD, and found that high plasma levels of hs-CRP (odds ratio [OR] 6.23, [95% CI, 1.93–20.12], *P* = 0.002), IL-6 (OR 13.63, [95% CI, 3.33–55.71]; *P* < 0.001), and TNF-α (OR 19.95, [95% CI, 4.93–80.78]; *P* < 0.001) were positively correlated with the incidence of myocardial injury (Table 3).

**Table 3:** Logistics regression for the association of inflammation with myocardial injury.

	**Univariate**			**Multivariate**		
	**OR**	**95% CI**	***P*-value**	**OR**	**95% CI**	***P*-value**
hs-CRP ≥ 86.5 mg/L	10.80	1.97–59.15	0.006	6.23	1.93–20.12	0.002
IL-2R ≥ 1041.0 pg/ml	3.81	0.86–16.94	0.079	2.23	0.71–7.02	0.17
IL-6 ≥ 703.9 pg/ml	9.13	2.92–28.50	<0.001	13.63	3.33–55.71	<0.001
IL-8 ≥ 29.4 pg/ml	7.27	1.35–39.05	0.021	2.53	0.84–7.58	0.098
TNF-α ≥ 13.8 pg/ml	17.36	3.04–99.20	0.001	19.95	4.93–80.78	<0.001

*OR, odds ratio; CI, confidence interval. hsCRP, high-sensitivity C-reative protein; IL, interleukin; IL-2R, interleukin-2 receptor; TNF-α, tumor necrosis factor α. Adjusted variates included age, smoking history, and pre-existing CVD*.

## Discussion

This study revealed that myocardial injury was associated with a high mortality rate in critically ill patients with COVID-19, and a high inflammatory burden was one of the potential causes of myocardial injury occurrence.

Of 64 in-ICU patients (42 males, 64.8 ± 12.2 years), 52 (81.5%) received invasive mechanical ventilation, and 47 (73.4%) died during the follow-up. A high incidence of COVID-19-induced myocardial injury was suggested by this study, since we found that 34 (53.1%) patients were diagnosed with myocardial injury, which is much higher than the incidence of myocardial injury in non-ICU patients (7.2% to 37.5%) (1, 4, 5). Myocardial injury usually contributes to various CV complications, such as cardiac dysfunction, arrhythmias and sudden death in patients with viral infectious diseases, which are associated with adverse events and a high mortality rate (13, 14). Several current studies have demonstrated that myocardial injury is associated with fatal outcomes and high mortality rates in hospitalized patients with COVID-19 (3, 5). In our study, in-ICU patients with myocardial injury were more likely to have preexisting cardiovascular diseases, develop cardiovascular complications, have higher APACHE-II/SOFA scores and have increased in-ICU mortality. In addition, Cox regression analysis suggested that myocardial injury was an independent risk factor for mortality, supporting that myocardial injury was associated with adverse events and high mortality rate in COVID-19 patients with critical illness.

Patients with COVID-19 were revealed to have a high systemic inflammatory status (1, 8). To date, the high systemic inflammation in hospitalized COVID-19 patients has been speculated as one of the potential causes of myocardial injury, as investigators found that hs-CRP levels positively correlated with plasma troponin levels in patients with COVID-19 (4). A similar finding was shown in our study. In addition to plasma hs-CRP, plasma levels of IL-1β, IL-2R, IL-6, IL-8, IL-10, and TNF-α were analyzed in this study, and IL-2R, IL-6, IL-8, and TNF-α were significantly increased in myocardial injury patients (Table 2). Moreover, patients with the high inflammatory burden were also shown to more likely develop myocardial injury (Figure 3). After univariate and multivariate logistic regression, the levels of hs-CRP, IL-6, and TNF-α were shown to be positively correlated with the incidence of myocardial injury, supporting the hypothesis that a high systemic inflammatory burden might contribute to myocardial injury in COVID-19 patients.

Hypoxemia, septic shock, coagulation disorders and cardiac arrhythmias are potentially involved in the process of systemic high inflammatory burden-induced myocardial injury in patients with severe acute respiratory syndrome coronavirus 2 (SARS-CoV-2) infection (15–17). These pathophysiological disorders were further illustrated by our study. In our patients, more than 95% of them developed ARDS with a significantly rapid heart rate, which caused an imbalance between cardiac metabolic demand and oxygen supply. Moreover, a prevalence of shock or insufficient peripheral perfusion was indicated by the common application of vasoconstrictive agents in our patients. Concomitantly, coagulation disequilibrium (higher D-dimer levels and longer PT) and the incidence of CV complications, including arrythmias, were also widely found in these patients. Myocardial inflammation might be another cause of myocardial injury in coronavirus-infected patients (15). In a study performed with 21 autopsies of SARS-CoV infected patients, Oudit et al. reported increased inflammation in the myocardium of these patients associated with cardiac interstitial fibrosis and hypertrophy (18). For COVID-19, a brief case report suggested the potential occurrence of myocarditis in COVID-19 patients by describing a 53-year-old woman diagnosed with COVID-19 who developed acute myocarditis during hospitalization (19). However, COVID-19-induced viral myocarditis has not been supported by pathological data so far. In current autopsy reports of COVID-19 patients, researchers revealed that there was only a mild infiltration of inflammatory cells without substantial necrosis of cardiomyocytes (20, 21). It seems that systemic inflammation, but not localized myocardial inflammation plays a pivotal role in myocardial injury of COVID-19 patients. A similar conclusion was also given by another retrospective study that enrolled 112 COVID-19 patients, as they revealed that there were no typical signs of myocarditis on echocardiography, such as segmental wall motion abnormality, reduced LVEF or wall thickening, in COVID-19 patients with myocardial injury during hospitalization (5). The roles of myocardial inflammation in myocardial injury still need more investigation.

The mechanisms underlying the activation of inflammation in COVID-19 patients have been recently investigated by Zhang et al. (22). It has been suggested that COVID-19 induced the destruction of alveolar epithelial cells, which led to an increase in cell permeability and the release of virus. This result subsequently activated the innate immune system and induced the overproduction of cytokines (e.g., IL-6 and TNF-a), finally causing a systemic inflammatory response (22, 23). In this process, macrophage recruitment, which has been demonstrated to regulate SARS-CoV-2-induced inflammation (24, 25), was suggested to be one of the potential contributors, as interstitial mononuclear inflammatory infiltrates were observed in both lungs of patients with COVID-19. In addition, the plasma levels of macrophage-produced pro-inflammatory cytokines, such as IL-6 (26) and TNF-α (27), were shown to be significantly increased in patients with COVID-19, further supporting the contribution of macrophages to systemic inflammation in COVID-19 patients. In addition to macrophages, the activation of lymphocytes was also suggested as a factor in systemic inflammation in COVID-19 patients (6). Although decreased blood lymphocyte count of COVID-19 patients has been widely reported (4, 28), lymphocytes were shown to be activated as the increases in the expression of HLA-DR in CD4^+^ and CD8^+^ cells, the percentage of CD4^+^ CCR4^+^ CCR6^+^ Th17 cells. The expression of cytotoxic particles (e.g., perforin and granulysin) in CD8 + T cells was demonstrated in an autopsy report of COVID-19 patients (21).

The efficiencies of anti-inflammatory treatments such as glucocorticoids, tocilizumab (TCZ) and anti-TNFα agents in COVID-19 patients were recently investigated by various registered cohort studies (6, 29). In this study, glucocorticoids and TCZ were applied in these patients. Glucocorticoids were widely applied during the outbreaks of several viral infectious diseases, such as SARS-CoV (30), Middle East respiratory syndrome (MERS)-CoV (31) and influenzas (32). However, the benefit derived from corticosteroids in the treatment of these diseases has not been revealed (33). For COVID-19, there are no clinical data indicating the benefits of corticosteroids, and the recommendation for their use is controversial (6, 33). Other investigators held a positive opinion for glucocorticoid usage, as systematic corticosteroid therapy in the first 3–5 days was shown to effectively inhibit severe inflammatory storms and alleviate critical symptoms in ICU patients with MERS (34). Currently, short-term systematic corticosteroid treatment (methylprednisolone, <1–2 mg/kg/d, 3–5 days) is recommended for the treatment of selective severe COVID-19 patients while being cautious of glucocorticoid-mediated immunosuppression, which delays the clearance of SARS-CoV-2 (35). In our study, systemic corticosteroid administration (methylprednisolone, 1–2 mg/kg/d × 5–7 days) was empirically used for patients with high inflammatory status. However, the efficiency is still not confirmed. Elucidating the benefit of glucocorticoids for COVID-19 patients is of immediate clinical importance. In contrast to glucocorticoids, TCZ, a recombinant human IL-6 monoclonal antibody, showed potential therapeutic value for COVID-19 patients. In a current clinical trial (clinical trial registration ID: ChiCTR2000029765), TCZ was administered once to 21 critical patients with COVID-19 at 400 mg intravenously. After a few days, the febrile patients' body temperature returned to normal, and all other symptoms improved significantly, in conjunction with better respiratory function, absorbed pulmonary lesions and lower plasma levels of hs-CRP (36). Moreover, several recent case reports also described the successful use of TCZ treatment in COVID-19 patients combined with other diseases (37, 38). In our study, TCZ was applied in several selective patients with high IL-6 levels. However, due to the insufficiency of related evidence, guidance and specialist consensus for the application of TCZ in COVID-19 patients is still lacking. Studies with larger populations are expected to further confirm the therapeutic value of TCZ against COVID-19 development. TNF-α inhibitors, such as infliximab (Remicade) and adalimumab (Humira), were not applied in our patients due to the lack of related information in COVID-19 patients. However, the therapeutic value of TNF-α for the severe immune-based pulmonary injury caused by SARS coronavirus has been implicated (39). Since high plasma levels of TNF-α have been widely observed in our patients, it is worth investigating the effects and safety of TNF-α inhibitors in the treatment of COVID-19.

This study still has several limitations. First, several pieces of cardiac information, such as echocardiography data and electrocardiography data, were lacking in this study, which limited the evaluation of myocardial injury. Second, plasma levels of certain inflammatory cytokines, such as granulocyte-colony stimulating factor, monocyte chemoattractant protein-1 and macrophage inflammatory protein 1-α (chemokine ligand 3), were not tested in our study. Finally, this study only involved 64 patients, and further studies with larger populations or multicenter study should be performed to confirm our results.

## Conclusion

This study demonstrated that myocardial injury was a common complication of COVID-19, and myocardial injury was associated with the occurrence of adverse events and a high mortality rate. The positive correlation of high inflammatory burden with the incidence of myocardial injury was further revealed in critically ill patients with COVID-19 in this study.

## Data Availability Statement

The datasets presented in this article are not readily available because the data which has been used is confidential. Requests to access the datasets should be directed to the corresponding author, camsww@163.com.

## Ethics Statement

The study protocol conforms to the ethical guidelines of the 1975 Declaration of Helsinki and was approved by the Ethics Committee of PUMC Hospital. Written informed consent was waived due to the rapid emergence of this infectious disease.

## Author Contributions

YS, PG, TR, WW, and SZ: concept and design. YS, PG, TR, HQ, FG, LC, and WW: acquisition, analysis, or interpretation of data. YS, WW, and SZ: drafting of the manuscript. YS, PG, TR, HQ, FG, LC, WW, and SZ: critical revision of the manuscript for important intellectual content. YS and WW: statistical analysis. WW and SZ: administrative, technical, material support, and supervision. All authors contributed to the article and approved the submitted version.

## Conflict of Interest

The authors declare that the research was conducted in the absence of any commercial or financial relationships that could be construed as a potential conflict of interest.

The handling editor is currently organizing a Research Topic with one of the authors SZ, and confirms the absence of any other collaboration.
